# Defining microbial community functions in chronic human infection with metatranscriptomics

**DOI:** 10.1128/msystems.00593-23

**Published:** 2023-10-12

**Authors:** Aanuoluwa E. Adekoya, Hoody A. Kargbo, Carolyn B. Ibberson

**Affiliations:** 1 Department of Microbiology and Plant Biology, University of Oklahoma, Norman, Oklahoma, USA; University of Illinois at Chicago, Chicago, Illinois, USA

**Keywords:** human infection, transcriptomics, chronic wounds, cystic fibrosis, microbial community functions

## Abstract

Chronic polymicrobial infections (cPMIs) harbor complex bacterial communities with diverse metabolic capacities, leading to competitive and cooperative interactions. Although the microbes present in cPMIs have been established through culture-dependent and culture-independent methods, the key functions that drive different cPMIs and the metabolic activities of these complex communities remain unknown. To address this knowledge gap, we analyzed 102 published metatranscriptomes collected from cystic fibrosis (CF) sputum and chronic wound infections (CW) to identify key bacterial members and functions in cPMIs. Community composition analysis identified a high prevalence of pathogens, particularly *Staphylococcus* and *Pseudomonas*, and anaerobic members of the microbiota, including *Porphyromonas*, *Anaerococcus*, and *Prevotella*. Functional profiling with HUMANn3 and SAMSA2 revealed that while functions involved in bacterial competition, oxidative stress response, and virulence were conserved across both chronic infection types, >40% of the functions were differentially expressed. Higher expression of antibiotic resistance and biofilm functions was observed in CF, while tissue-destructive enzymes and oxidative stress response functions were highly expressed in CW samples. Of note, strict anaerobes had negative correlations with traditional pathogens in both CW and CF samples, and they significantly contributed to the expression of these functions. Additionally, we show that microbial communities have unique expression patterns, and distinct organisms fulfill the expression of key functions in each site, indicating that the infection environment strongly influences bacterial physiology and that community structure influences function. Collectively, our findings indicate that community composition and function should guide treatment strategies for cPMIs.

**IMPORTANCE:**

The microbial diversity in polymicrobial infections (PMIs) allows for community members to establish interactions with one another, which can result in enhanced disease outcomes such as increased antibiotic tolerance and chronicity. Chronic PMIs result in large burdens on health systems, as they affect a significant proportion of the population and are expensive and difficult to treat. However, investigations into physiology of microbial communities in actual human infection sites are lacking. Here, we highlight that the predominant functions in chronic PMIs differ, and anaerobes, often described as bystanders, may be significant in the progression of chronic infections. Determining the community structure and functions in PMIs is a critical step toward understanding the molecular mechanisms that increase the virulence potential of the microbial community in these environments.

## OBSERVATION

Microbes live in multi-species communities where community structure and function dictate key processes such as nutrient cycling, tolerance to disturbances, and disease progression in infection sites. The presence of diverse microbes with a wide range of metabolic capacities and large nutrient gradients often leads to microbe-microbe interactions in chronic polymicrobial infections (cPMIs), which dictate overall community function and impact disease progression (1–3). However, although we have known that chronic infections are composed of polymicrobial communities for over 100 years, pathogenesis research has focused on the physiology of a handful of well-known pathogens in isolation *in vitro* and in animal models, and data on microbial community physiology in human infection sites are lacking (4–6). Furthermore, the contribution of the normal flora identified in cPMIs to disease progression has remained debatable, and members of the microbiota are often ignored in current treatment plans (7). Therefore, two important knowledge gaps are the key functions that drive each chronic PMI and the metabolic activities of the array of microbes present. To address these questions, we analyzed 102 previously published metatranscriptomes collected from people with cystic fibrosis (CF) (30%) and chronic wound (CW) infections (70%) to identify key bacterial members and community functions in these typical examples of clinically important cPMIs (8, 9).

### Anaerobes are prominent in chronic infections

**Fig 1:**
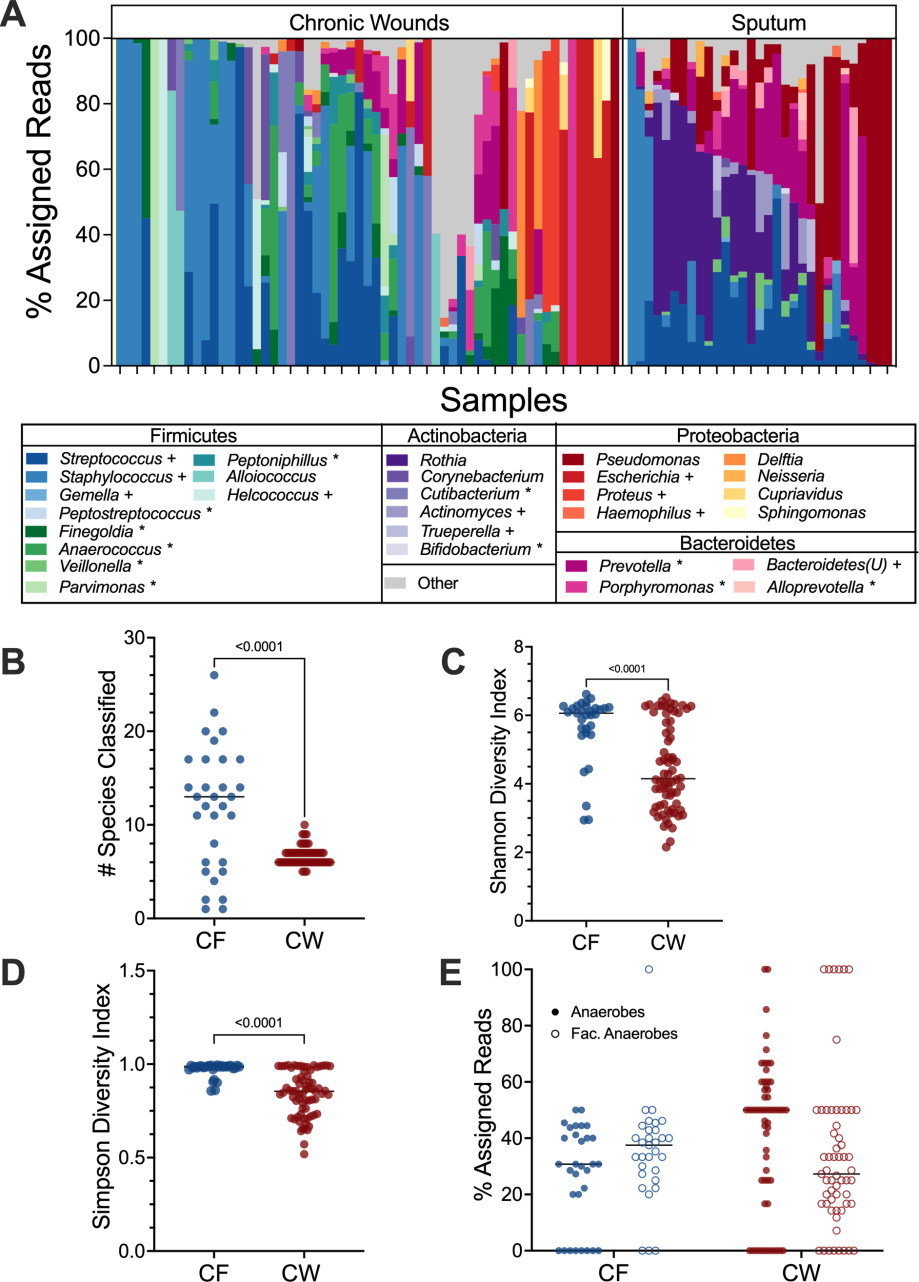
Bacterial community composition in CF and CW environments. (**A**) Relative abundance of bacterial genera present in at least three samples with a % assigned read abundance of at least 1%. 26 unique genera were identified in CF samples and 36 in CW samples. “*Bacteroidetes(U)*” were members of the phylum Bacteroidetes that lacked specific genus level classification. “Others” were genera identified at a relative abundance less than 1% and in fewer than three samples. (**B**) Distribution of the number of species with a relative abundance of at least 1% in CF and CW samples. (**C**) The Shannon diversity index of each sample. (**D**) Distribution of the Simpson diversity index of each sample. (**E**) Distribution of the percentage of reads assigned to anaerobes (closed circles) and facultative anaerobes (open circles) in each sample in the CF and CW environments. For plots B–E, CF samples are in blue and CW samples are in red. *P* values and brackets indicate comparisons that were deemed statistically significant.

We identified transcriptionally active microbial communities in 90 of our 102 samples (CF: 31, CW: 59) through community composition analysis with MetaPhlAn4 (Data Set S1). Identification of the transcriptionally active genera present revealed that both the CW and CF sputum samples contained a mix of traditional pathogens from the genera *Staphylococcus*, *Pseudomonas*, and *Streptococcus*, along with anaerobic members of the microbiota (Fig. 1A)*,* concordant with what is expected in these infections based on previous metagenomic and 16S rRNA gene data (8, 10–13). Of note, the *Corynebacterium* and *Streptococcus* species identified in our samples were almost exclusively known pathogenic species (*Corynebacterium striatum*, *Streptococcus pneumoniae*, *Streptococcus agalactiae*, and *Streptococcus mitis*). While the mean number of species identified in each sample aligns with previous reports (10, 12–15), we found the CF samples were more diverse than CW samples, with a mean of 11.8 and 6.7 species identified, respectively (*P* value <0.01) (Fig. 1B). The increased diversity in CF sputum compared to CW wounds was also observed with both Shannon and Simpson diversity indices (Fig. 1C and D). Interestingly, we identified a high abundance of transcripts assigned to anaerobes in these samples (Fig. 1A and E), suggesting that chronic infection environments are likely hypoxic. Furthermore, we found that while anaerobes co-occurred with traditional pathogens in over 50% of samples (CF: 80.7%, CW: 52.5%), there was a strong negative correlation between the anaerobes and traditional pathogens in both sites, indicating possible competitive interactions (Fig. S1). It should be noted that we used metatranscriptomes to assess the transcriptional activity for these analyses as metagenomes and 16S rRNA sequences were not available for all samples. This distinction is an important consideration when evaluating the differences in alpha-diversity here. Furthermore, the samples used for this analysis were previously published data from different sources, and it is possible that there are differences in sample collection methods or contamination with members of the skin and oral microbiota. However, the congruence in our findings across studies and the high prevalence of reads from the microbiota make these possibilities unlikely.

### CF sputum has increased expression of antibiotic resistance and biosynthetic pathways, while tissue-destructive and catabolic pathways are primarily expressed in CW infections

**Fig 2:**
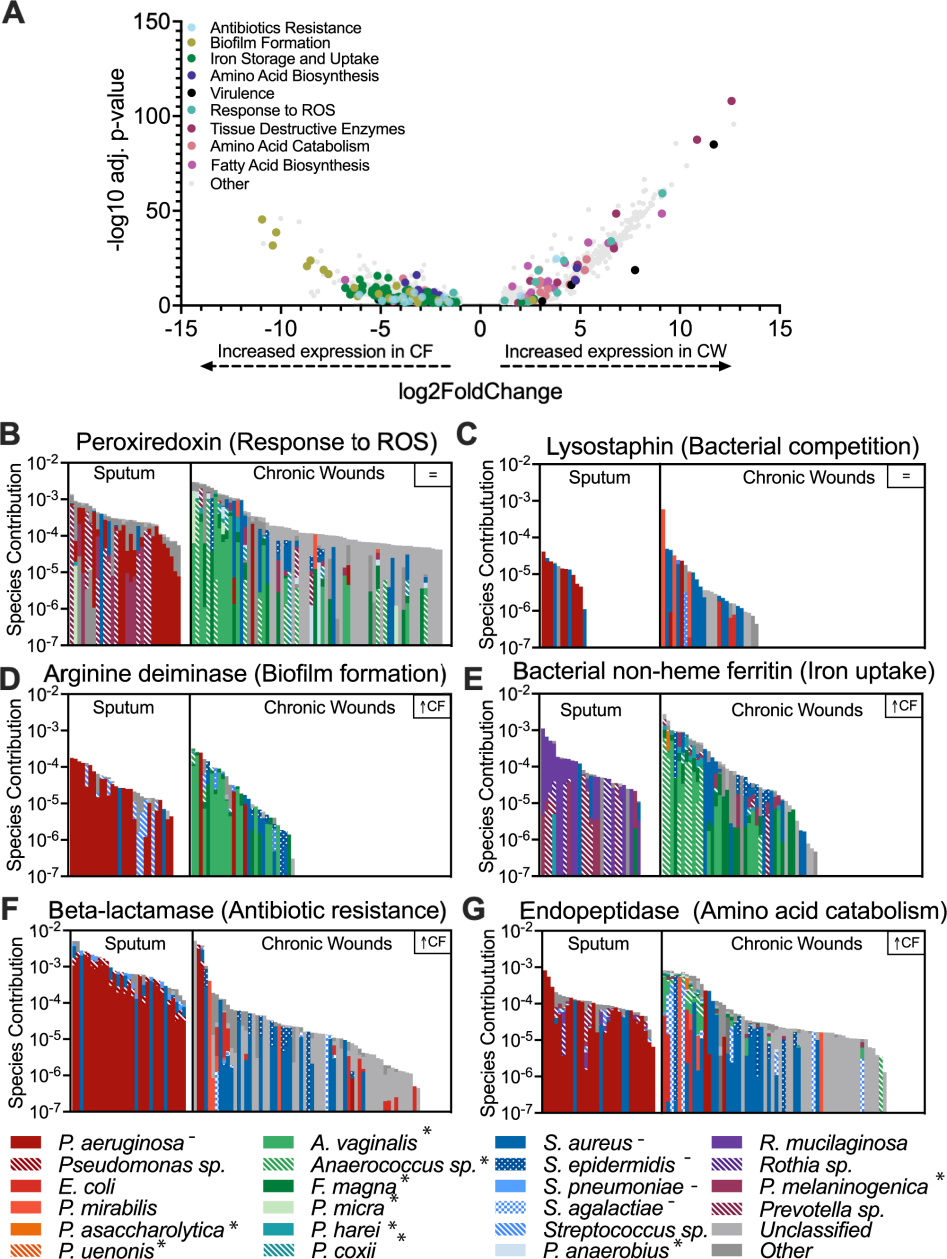
Distinct expression of microbial functions in CF and CW communities. (**A**) Volcano plot to highlight differentially expressed functions between infection sites as identified by SAMSA2. Of the functions, 40.37% were differentially expressed (adjusted *P* value < 0.05, log2 fold change >1 with 1,335 and 488 functions highly expressed in CF and CW samples, respectively. (**B and C**) Bacterial contribution to the expression of functions conserved across CF and CW environments. (**D–G**) Bacterial contribution to the expression of differentially expressed functions. Species contribution (*y*-axis) refers to the sum of the relative abundance of the bacterial species per sample for each function in log scale.

Through profiling with both SAMSA2 and HUMANn3, we classified the level 4 enzyme commission (EC) functions in each sample (SAMSA: 4527, HUMANn3: 2459). Our analysis revealed that several EC classes involved in oxidative stress responses, virulence, bacterial competition, fatty acid metabolism, and iron acquisition were not differentially expressed across infection environments (Data Set S2, sheet 1), indicating that bacterial community members in these infection types may be competing with one another for resources while tolerating host innate immune mechanisms and simultaneously expressing their virulence functions. However, while some key functions were conserved across both infection sites, over 40% of the functions identified were differentially expressed (*q* value <0.05, fold change >2) between the two sites (40.4% and 43.0% for SAMSA2 and HUMANn3, respectively), and there were key differences in the types of functions that were highly expressed in each site. CF sputum displayed high expression of antibiotic resistance functions, iron acquisition, virulence factors, and functions important for attachment to host surfaces (Fig. 2; Fig. S2 and S3; Data Set S2, sheets 2–5). In contrast, CW infections had high expression of functions involved in oxidative stress response and tissue-destructive enzymes (Fig. 2; Fig. S2 and Data Set S2, sheets 2–5). Taking a deeper look into the expression of metabolic pathways in each site revealed the enrichment of catabolic pathways, such as the glycogen degradation pathway and the valine degradation pathway, as well as catabolism support pathways, including phospholipase synthesis, in CW samples (Data Set S3). In contrast, in the CF samples, there was an enrichment of biosynthetic pathways, such as the fatty acid elongation pathway, oleate, palmitoleate, and valine biosynthesis.

The increased expression of functions involved in multiple classes of antibiotic resistance in sputum strongly suggests that bacterial community in CF airways may have adapted to negating the effect of the antibiotics used in the management of infection, possibly contributing to the persistence of the lung infection. Furthermore, the enrichment in biosynthetic pathways and siderophores in CF lung communities indicates these key nutrients are likely limited in this environment and these results align with previous studies that have explored bacterial metabolic activities and nutrient composition in human CF sputum (6, 11). In contrast, the high expression of oxidative stress response, tissue-destructive enzymes, and catabolic pathways in CW infections indicates the complex community in these infections is degrading host tissue to release nutrients and that nutrients are likely abundant, possibly contributing to bacterial virulence and persistence. This may also be due to the high presence of *Staphylococcus aureus* in CW infections, which is notorious for synthesizing large quantities of tissue-destructive enzymes (16). The shift in the key functions identified in each infection site suggests that the microbial community is highly responsive to the infection environment, where environmental cues and the nutritional landscape are key drivers of microbial physiology.

### Bacterial community structure and environment influence function

In addition to the distinct functions identified in each infection site, we were interested in if the same or distinct community members were contributing to each site. Therefore, we analyzed the stratified output provided by HUMANn3 to evaluate community member contributions. We observed that transcripts were frequently assigned to common pathogens such as *Pseudomonas aeruginosa*, *Staphylococcus epidermidis*, *S. aureus*, *Streptococcus agalactiae*, and anaerobic members of the microbiota such as *Anaerococus vaginalis*, *Finegoldia magna, Prevotella melaninogenica*, and *Veillonella parvula*. While both groups were prominent contributors to the reduction of oxidative stress and bacterial competition, iron acquisition and biofilm functions were mostly expressed by *P. aeruginosa* in the CF environment, while *S. aureus* dominated expression in CW infections. Additionally, tissue-degrading enzymes were primarily expressed by *P. aeruginosa* in CF sputum but by the anaerobic microbiota in CW infection (Fig. 2; Fig. S3). Taken together, our data show that key community functions are expressed by distinct species in each site, indicating niche differentiation may be occurring during chronic infection. However, it should be noted that one limitation is that the short reads used may not allow for species-level identification of all functions by HUMANn3.

### Conclusions and key takeaways

We found that the key functions that drive disease progression in each infection type differ. Furthermore, we showed that the microbial community in each infection type is distinct, and this compositional difference, alongside the infection environment, is critical in determining functions important for disease progression. Interestingly, we found that the anaerobic microbiota may play a significant role in the progression of chronic infections. Together, these findings will prompt future studies aimed at investigating how co-infecting microbes interact with traditional pathogens, the molecular mechanisms that drive these interactions, and how these interactions impact bacterial virulence and disease chronicity.

## MATERIALS AND METHODS

### Data set collection and validation

We analyzed 102 RNA-sequencing files of chronic wound and cystic fibrosis patients from published studies (7, 11, 16–19). We limited our search to metatranscriptomes collected from people with CW in lower extremities and CF and ensured the absence of technical replicates or transcriptomes with reads previously mapped to single bacterial species, which identified six studies that fit these criteria. We assessed the quality of the sequence files using FastQC version 0.11.9 (20) and removed adapter sequences and reads less than 22 bases with CutAdapt version 4.1 (21). Ribosomal RNA sequences were removed with SortMeRNA version 4.0.0 (22) using default parameters. The resulting reads were mapped to the human genome (GRCh38/hg38), and processed reads that did not map to GRCh38 were used for community and functional analyses.

### Metatranscriptome analysis

MetaPhlAn version 4.0.1 was used for community composition analysis and to obtain the relative abundances of bacteria in each sample using a minimum read length threshold of 22 bases and other default parameters (23). SAMSA version 2.0 and HUMAnN version 3.0 were used for functional profiling. First, we analyzed the prokaryotic non-rRNA reads with SAMSA2 to identify the functional profile of the microbial community in each sample (24). SAMSA2 annotated the reads against the RefSeq bacterial database and SEED subsystems database using DIAMOND aligner. Outputs were aggregated and exported for statistical analysis with DESeq2 version 1.38.3 in RStudio. In addition, we also did functional profiling with HUMAnN3 to obtain the metabolic potential of the microbial communites (25). HUMANn3 uses the DIAMOND aligner to map reads to the UniRef90 database to identify the UniRef protein families, which were regrouped to level 4 ECs. We normalized the reads per kilobase output from HUMAN3 to relative abundance data with the humann_renorm_table script, and the data was used as input in MaAsLin2 version 1.12.0 for statistical analysis in RStudio. The metabolic feature of HUMANn3 was also used to map the reads to MetaCyc pathways.

### Statistical analyses

All other statistical analyses were performed in RStudio with R version 4.2.2. Data visualizations were performed in GraphPad Prism version 9.

## Data Availability

All codes used in these analyses are available at https://github.com/Aanuoluwaduro/Metatransriptomics-Microbial-Community-Functions. The 102 metatranscriptomes used in this study were pulled from the National Center for Biotechnology Information Sequence Read Archive under accession numbers SRP135669, PRJNA573047, PRJNA563930, PRJNA726011, PRJNA576508, PRJNA720438, and PRJNA909326. Additional detailed methods are included in the supplemental material.

## SUPPLEMENTAL MATERIAL

Data Set S1 msystems.00593-23-s0001.xlsx Metadata for all samples used in this study.

Data Set S2 msystems.00593-23-s0002.xlsx Differentially expressed functions as identified by SAMSA2 and HUMANn3.

Data Set S3 msystems.00593-23-s0003.xlsx Differentially expressed pathways identified by HUMANn3.

Supplemental Material msystems.00593-23-s0004.pdf Additional methods; Figures S1 to S3; captions for Data Sets S1 to S3.

ASM does not own the copyrights to Supplemental Material that may be linked to, or accessed through, an article. The authors have granted ASM a non-exclusive, world-wide license to publish the Supplemental Material files. Please contact the corresponding author directly for reuse.
