# Supplementary material for: Defining microbial community functions in chronic human infection with metatranscriptomics
Source: mSystems. 2023 Oct 12;8(6):e00593-23. doi: 10.1128/msystems.00593-23 (PMC10734476; doi:10.1128/msystems.00593-23)
Supplement: Supplemental Material — Additional methods; Figures S1 to S3; captions for Data Sets S1 to S3. [file msystems.00593-23-s0004.pdf]

Supplementary Material

Materials and Methods

Dataset Collection and Validation: The datasets used for this study were identified by body part or tissue being affected in samples collected – chronic lower extremity ulcers for CW samples and sputum samples from people with CF, presence of original metatranscriptomic data, presence of comorbidities in patients and treatment course. We passed only reads with a minimum length of 22 to improve the specificity of mapping to reference databases in downstream application as previous data has shown that read length influences mapping (1).

Community Composition Analysis: We reduced our Metaphlan4 output data to genera and species that had at least one percent abundance prevalence in at least 3 samples. Then we grouped these organisms at both genera and species levels as either aerobes, facultative anaerobes or obligate anaerobes. We defined obligate/facultative anaerobes based on culture requirements and how these organisms have frequently been identified/grouped in previous literature and textbooks (2-9).

Functional Profiling: Two bioinformatic tools were used for functional profiling. The Simple Annotation of Metatranscriptomes by Sequence Analysis tool **(**SAMSA2) identified 4527 level 4 enzyme classes and the DIAMOND_analsys_counter.py scripts were used to aggregate the outputs which were eventually exported to R studio for statistical analysis. The diversity_stats.R script of the SAMSA2 package was used to compute the mean Shannon and Simpson diversity indices for the two infection communities and the diversity_gaphs.R script was used to plot the graphs of these. We performed differential expression analysis of the features in the both communities using the run_DESeq_stats.R script. The HMP Unified Metabolic Analysis Network (HUMAnN3) tool, identified 594,273 UniRef protein families which were further regrouped to 2459 unique ECs using the humann_regroup_table command and exported to Rstudio for differential expression analysis. For pathway mapping by HUMANn3, 490 pathways with metacyc IDs were identified and 162 of these had differential expression across the two infection communities.

Assigning Functions to Functional Categories: To assign E.C. numbers to functional categories, we first converted E.C. numbers to KEGG terms and used these to assign them to KEGG pathways. Pathways were then assigned to broader unified functional categories (e.g. Histidine metabolism and Tryptophan metabolism were both reclassified to “Amino acid metabolism”). Any functions without a KEGG ID were annotated with the ExplorEnz, BRENDA, and InterPro databases if possible. Pathways were assigned to MetaCyc “superclasses” based on their pathway IDs and then added to unified functional categories if necessary (e.g. peptidoglycan biosynthesis and lipid A biosynthesis were both assigned to “Cell Wall & Cell Membrane biosynthesis”). While E.C classes allowed us to perform functional category assignment, some of the additional identified features from SAMSA2 were not assigned E.C numbers or were not enzymes such as flagella, pili, typeIII secretion systems, and these were assigned functional categories based on description in previous literature (10-12).

Statistical Analyses:

For the HUMANn3 functional analyses, we performed differential expression analysis using DESeq2 1.38.3 and MaAsLin2 1.12.0 in R studio.

Figures

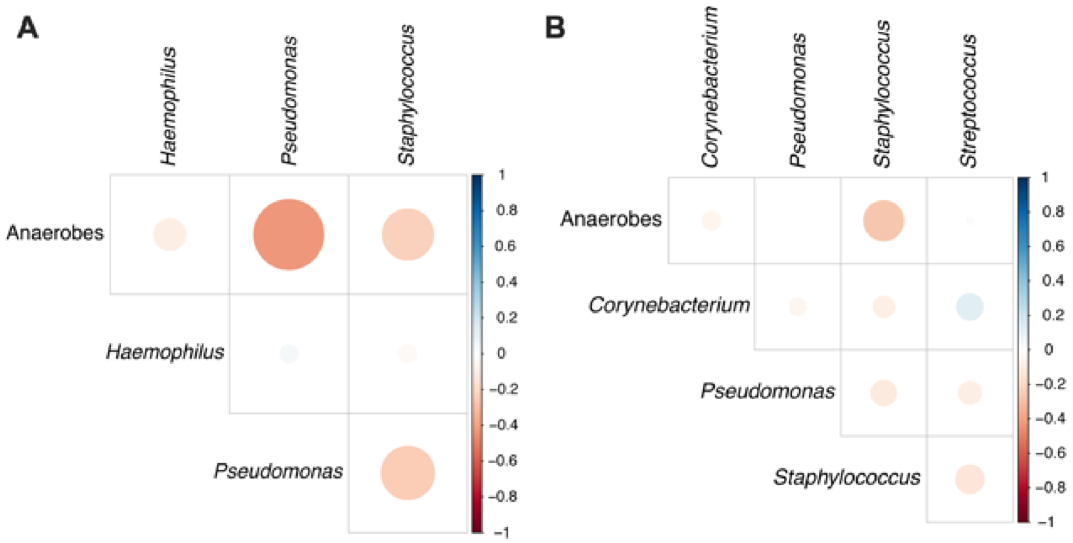

Figure S1: Co-occurrence of obligate anaerobes and traditional pathogens in CF and CW samples. **A)** Obligate anaerobes (*Prevotella*, *Actinomyces*, *Veillonella* & *Fusobacterium*) have negative correlation with CF pathogens (*Pseudomonas*, *Staphylococcus*, & *Haemophilus*). **B)** Obligate anaerobes (*Finegoldia, Anaerococcus*, *Peptoniphilus*, *Peptostreptococcus*, *Parvimonas* and *Peptococcus*) have negative correlation with CW pathogens (*Staphylococcus*, *Streptococcus*, *Pseudomonas*, and *Corynebacterium*). **A&B** show the Pearson correlation coefficients between the sum of the relative abundances of the anaerobes, and each indicated pathogen. The size of the circles corresponds to their Pearson correlation coefficients while the color denotes if they are positive (blue) or negative (red).

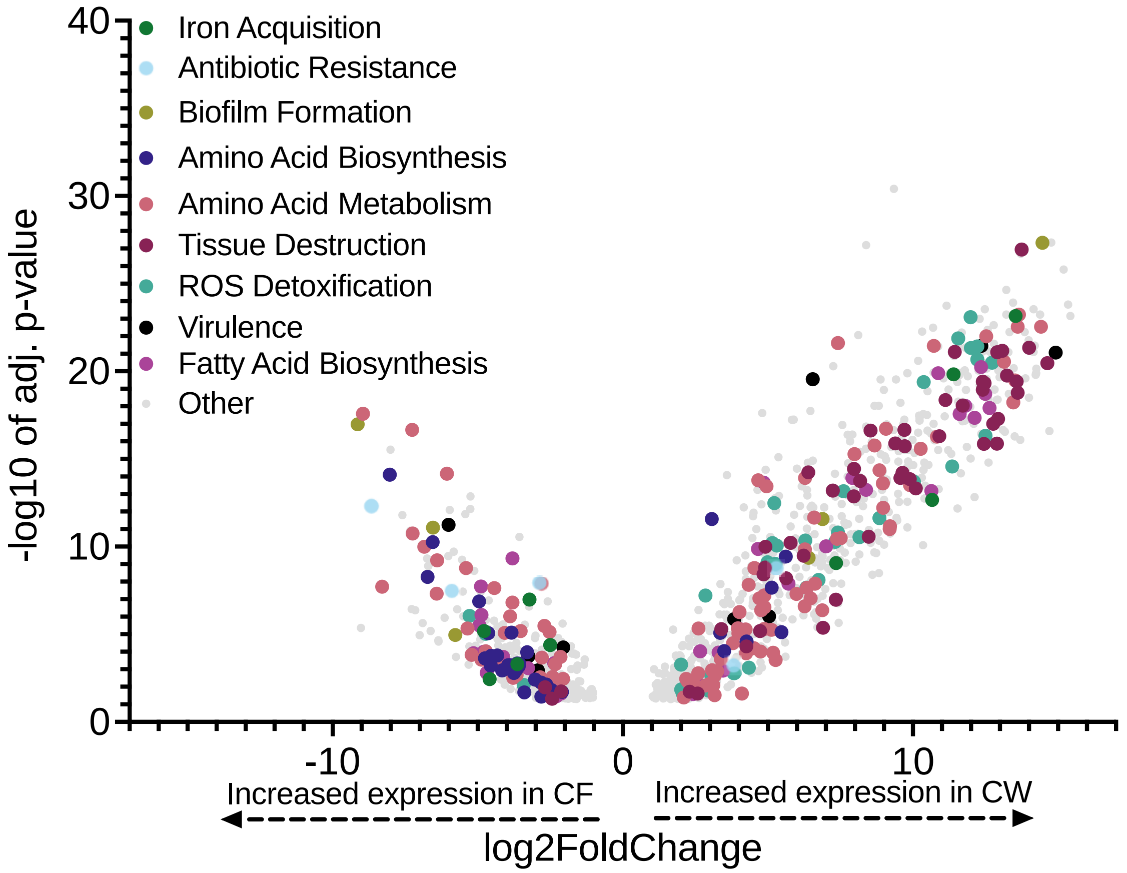

Figure S2: Volcano plot to highlight differentially expressed functions in both infection communities as identified by HUMANn3. 42.98% of the functions were differentially expressed (adjusted p-value <0.05, log2FoldChange > 1) with 753 and 303 functions highly expressed in CW and CF samples respectively.

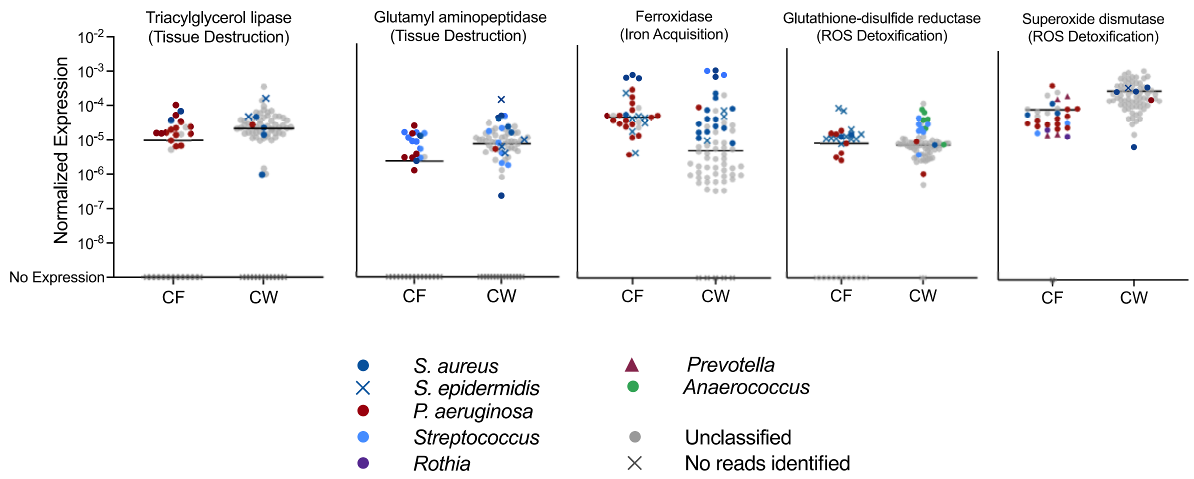

Figure S3: Dot plot to highlight bacterial contribution to the expression of differentially expressed functions in CF and CW environments. Normalized expression (y-axis) refers to the sum of the relative abundance of the bacteria species per sample for each function in log scale.

Supplementary Datasets

Dataset S1: Detailed metadata on all samples used for the study.

Dataset S2: Functional analysis data. Sheet 1 - highlighted conserved functions identified with HUMANn3 and SAMSA2 and bacterial contribution to these functions. Sheet 2 - highlighted differentially expressed functions identified with HUMANn3 and SAMSA2 and bacterial contribution to these functions. Sheet 3 - all differentially expressed functions obtained from HUMANn3. Sheet 4 - all differentially expressed functions obtained from SAMSA2.

Dataset S3: Pathway analysis data. Sheet 1- all pathway analysis data as identified by HUMANn3. Sheet 2 - all differentially expressed pathways obtained from HUMANn3.

SUPPLEMENTAL REFERENCES

1. Lewin, G. R., Stocke, K. S., Lamont, R. J., & Whiteley, M. (2022). A quantitative framework reveals traditional laboratory growth is a highly accurate model of human oral infection. Proceedings of the National Academy of Sciences, 119(2), e2116637119. https://doi.org/10.1073/pnas.2116637119
2. Boyanova, L., Vladimir Djambazov, Gergova, G., Dragomir Iotov, Petrov, D., Osmanliev, D., Minchev, Z., & Mitov, I. (2004). Anaerobic microbiology in 198 cases of pleural empyema: A Bulgarian study. Anaerobe, 10(5), 261–267. https://doi.org/10.1016/j.anaerobe.2004.06.001
3. Brook, I. (1986). Encapsulated anaerobic bacteria in synergistic infections. Microbiological Reviews, 50(4), 452–457.
4. Brook, I., & Frazier, E. H. (1990). Aerobic and anaerobic bacteriology of wounds and cutaneous abscesses. Archives of Surgery, 125(11):1445–1451. doi:10.1001/archsurg.1990.01410230039007
5. Choi, Y., Banerjee, A., McNish, S., Couch, K. S., Torralba, M. G., Lucas, S., Tovchigrechko, A., Madupu, R., Yooseph, S., Nelson, K. E., Shanmugam, V. K., & Chan, A. P. (2019). Co-occurrence of Anaerobes in Human Chronic Wounds. Microbial Ecology, 77(3), 808–820. https://doi.org/10.1007/s00248-018-1231-z
6. Dowell, V. R. (1974). Laboratory methods in anaerobic bacteriology: CDC laboratory manual. Center for Disease Control.
7. Gulzar, M., & Zehra, A. (2018). Staphylococcus aureus: A brief review. International Journal of Veterinary Science and Research. https://doi.org/10.17352/ijvsr.000031
8. Hardie, J. M., & Whiley, R. A. (1995). The genus Streptococcus. In B. J. B. Wood & W. H. Holzapfel (Eds.), The Genera of Lactic Acid Bacteria (pp. 55–124). Springer US. https://doi.org/10.1007/978-1-4615-5817-0_4
9. Mirković, B., Murray, M. A., Lavelle, G. M., Molloy, K., Azim, A. A., Gunaratnam, C., Healy, F., Slattery, D., McNally, P., Hatch, J., Wolfgang, M., Tunney, M. M., Muhlebach, M. S., Devery, R., Greene, C. M., & McElvaney, N. G. (2015). The Role of Short-Chain Fatty Acids, Produced by Anaerobic Bacteria, in the Cystic Fibrosis Airway. American Journal of Respiratory and Critical Care Medicine, 192(11), 1314–1324. https://doi.org/10.1164/rccm.201505-0943OC
10. Klausen, M., Aaes-Jørgensen, A., Molin, S., & Tolker-Nielsen, T. (2003). Involvement of bacterial migration in the development of complex multicellular structures in

Pseudomonas aeruginosa biofilms. Molecular Microbiology, 50(1), 61–68. https://doi.org/10.1046/j.1365-2958.2003.03677.x

11. Klausen, M., Heydorn, A., Ragas, P., Lambertsen, L., Aaes-Jørgensen, A., Molin, S., & Tolker-Nielsen, T. (2003). Biofilm formation by Pseudomonas aeruginosa wild type, flagella and type IV pili mutants. Molecular Microbiology, 48(6), 1511–1524. https://doi.org/10.1046/j.1365-2958.2003.03525.x
12. Veetilvalappil, V. V., Manuel, A., Aranjani, J. M., Tawale, R., & Koteshwara, A. (2022). Pathogenic arsenal of Pseudomonas aeruginosa: An update on virulence factors. Future Microbiology, 17(6), 465–481. https://doi.org/10.2217/fmb-2021-0158
